# Conservation Potential of Abandoned Military Areas Matches That of Established Reserves: Plants and Butterflies in the Czech Republic

**DOI:** 10.1371/journal.pone.0053124

**Published:** 2013-01-09

**Authors:** Oldrich Cizek, Pavel Vrba, Jiri Benes, Zaboj Hrazsky, Jiri Koptik, Tomas Kucera, Pavel Marhoul, Jaroslav Zamecnik, Martin Konvicka

**Affiliations:** 1 Faculty of Science, University of South Bohemia, Ceske Budejovice, Czech Republic; 2 Biological Centre CAS, Institute of Entomology, Ceske Budejovice, Czech Republic; 3 Hutur NGO, Hradec Kralove, Czech Republic; 4 Daphne CR – Institut Aplikovane Ekologie, České Budějovice, Czech Republic; 5 Museum of Eastern Bohemia, Hradec Kralove, Czech Republic; Swedish University of Agricultural Sciences, Sweden

## Abstract

Military training generates frequent and irregular disturbance followed by succession, resulting in fine-scaled mosaics of ecological conditions in military training areas (MTAs). The awareness that MTAs may represent important biodiversity sanctuaries is increasing recently. Concurrently, changes in military doctrine are leading to abandonment of many MTAs, which are being brought under civilian administration and opened for development. We surveyed vascular plants in 43 and butterflies in 41 MTAs in the Czech Republic and compared the records with plants and butterfly records from 301 and 125 nature reserves, respectively. After controlling for effects of area, geography, and climate, we found that plant species richness was equal in the two land use categories; butterfly richness was higher in MTAs; reserves hosted more endangered plants and more endangered butterflies. Ordination analyses, again controlled for potential nuisance effects, showed that MTAs and reserves differed also in species composition. While specialist species of nationally rarest habitat types inclined towards the reserves, MTAs hosted a high representation of endangered species depending on either disturbed ground, or successionaly transient conditions. These patterns reflect the history of the national nature reserves network, and the disturbance-succession dynamics within MTAs. The conservation value of formerly army-used lands is increasingly threatened by abandonment, and conservationists should support either alternative uses mimicking army activities, or sustainable management regimes.

## Introduction

Space is the ultimate ecological resource, and habitat loss and degradation represent the major cause of species’ declines [1]. Protected areas, established to safeguard the habitats and species, seldom do so optimally with respect to area and numbers of species harboured, because the current state of national reserve systems resulted from complex histories of the conservation movement [2]. The historically oldest reserves protected scenic sites of purportedly pristine character; later on, reserves strived to protect representative collections of natural habitats, ideally hosting declining species. Only relatively recently, with accelerating biodiversity loss, it has been accepted that reserves should safeguard a maximum of biotic potentials of respective regions to allow for continuation of evolutionary processes [3] and future biodiversity restoration [4], [5]. This view calls for conserving, in addition to natural and seminatural [6] habitats, also locations heavily affected by humans, but hosting otherwise rare species or processes [7], [8], [9].

Medium-sized military training areas (MTAs) represent such heavily human-influenced but potentially valuable sites. With the cessation of Cold war, and ensuing changes in military doctrine (professionalization, lower reliance on heavy armour: [10]), armies in developed countries have abandoned many previously used training grounds, once existing near every garrison town across Europe, North America, and elsewhere. Whereas large training ranges covering hundreds to thousands square kilometres, whose biodiversity potential is increasingly accepted [11], [12], [13], usually remain under military administration, the small- to medium-sized MTAs, covering tends to hundreds hectares, are being gradually transferred into civilian administration. The exact number of such sites is difficult to obtain. Around 60 training grounds used by heavy armour, and several hundreds infantry training fields, had existed in Czech Republic alone until the 1990s, which allows assuming that thousands such sites could have existed across Europe.

Past use of these sites was characterised by a combination of intensive disturbance from such activities as shelling or heavy armour movements [14], [15], [16] on the one hand, and exclusion of intensive agriculture/forestry, plus limited public access, on the other hand [11]. The resulting disturbance-succession dynamics create highly heteroeneous patchy conditions, which may generate resources for rich arrays of species [17], [18]. Assuming that natural biotopes would be highly dynamic even in the absence of human activities [19], [20], and that a substantial proportion of currently declining species utilise early-successional and/or highly heterogeneous conditions in Europe [13], [21], [22], [23], it could be expected that army training sites should support exceptional biodiversity.

A recent survey of bird communities inhabiting abandoned MTAs in the Czech Republic showed that these areas are particularly valuable for declining open habitats species [24]. Abandonment imperils this value, because in absence of disturbances, open vegetation develops towards woody formations [25]. Abandonment also opens the sites for various forms of exploitation, including intensive silviculture or building development.

Here, we document the conservation value of abandoned MTAs for other two important model groups of organisms, vascular plants and butterflies. By comparing the species richness and the numbers of red-listed species and species assemblages composition of the two study groups in the Czech Republic MTAs versus nature reserves, we show that the species richness within MTAs matches, or even surpasses, species richness within the reserves. We also employ a multivariate ordination technique to illustrate the differences between the species assemblages existing in the reserves and in MTAs, showing that the differences in species composition between the two land use categories are at least partly atrributable to the history of their establishment, and to differnces in local disturbance regimes.

## Materials and Methods

### Reserves and Military Areas

In addition to national parks, the Czech Republic protected areas system includes about 2000 smaller locations in several categories of legal protection, collectively coined here as reserves. The main division is between the National reserves, viewed as the most valuable ones and administered by the central government (*n* = 221); and Regional reserves, administrated by local governments.

The military-administered lands include five large training ranges, each covering hundreds of km^2^ (not considered here); and approximately 200 smaller MTAs, none exceeding 1000 hectares, 60 of which were historically used by armoured units. While some of these smaller MTAs have existed since the 19th century, the majority of them were established in the years preceding, or shortly following, World War II, and all the studied ones were abandoned in the 1990s.

### Taxa Studied

Czech Republic flora is made up of approximately 2750 taxa of vascular plants [26]; the number is higher if ornamental plants, apomictic and critical taxa etc., are included. The national Red list [27] contains 1543 endangered taxa, ca. 60% of the flora.

For butterfly analysis, we merged butterflies proper (Hesperioidea and Papilionoidea), and day-active burnet moths (Zygaenidae). A total of 165 species of these groups currently occur in the country (144 butterflies, 21 burnet moths), of which 84 (76, 8) are considered endangered [28].

### The Data

#### Plants in reserves

**Figure 1:**
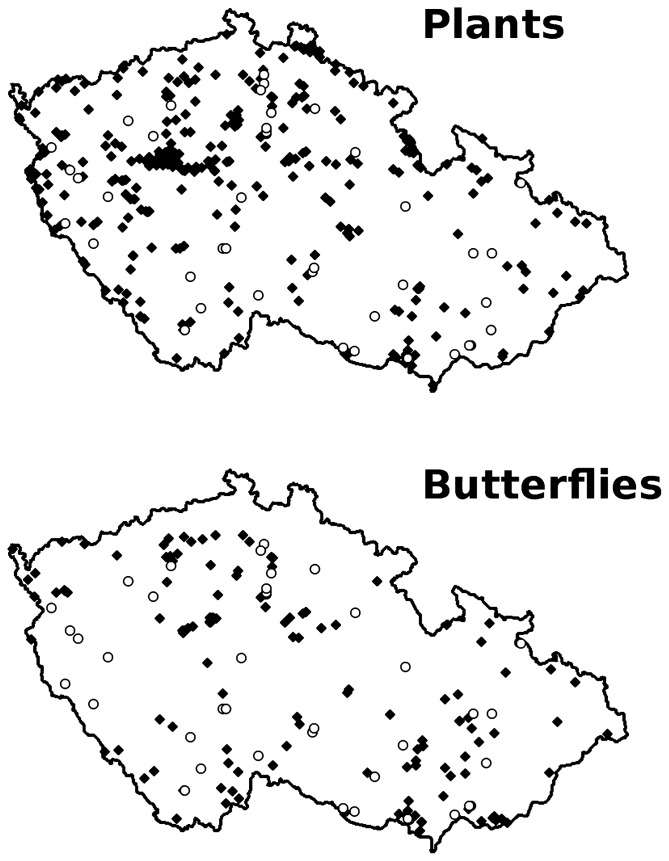
Maps of the Czech Republic showing positions of reserves (black diamonds) and military training areas (white circles) sites for vascular plants (top) and butterflies (bottom).

The data originated from reserve inventories, commissioned by the Czech Conservation Authority in the late 1990s. Skilled botanists, usually familiar with assigned sites, were asked to record as many as possible vascular plants species per reserve. A total of 301 thus surveyed reserves (Table 1, Fig. 1, Table S1) represent a sample of the Czech Republic reserve network balanced with respect to reserve areas, geographic locations or original conservation targets (e.g., vegetation, endangered species); see [29] for data acceptance criteria. *Endangered plants* were extracted from these lists.

**Table 1:** Overview of study sites characteristics, and available data, used to compare species richness, and numbers of endangered species, in the Czech Republic reserves and military training areas (MTAs).

	N	Area (ha)Mean ±SD (range)	Altitude (m)Mean ±SD (range)	T-M-O1)	Species total	Endangered species total	Species richnessper site	Endangered speciesper site
**MTAs**								
Plants	43	92±74.1 (21–351)	363±112.3 (200–625)	19–24-0	873	160	191±41.4 (50–251)	11±8.2 (0–45)
Butterflies	41	91±74.6 (21–351)	367±113.7 (200–625)	18–23-0	118	42	49±11.5 (15–70)	4±3.5 (0–13)
**All analysed reserves**								
Plants	301	117±392.0 (0.2–4279.8)	500±250.8 (150–1362)	78–154-63	1941	884	178±104.1 (17–593)	23±28.1 (0–165)
Butterflies	125	164±322.7 (0.3–2030)	437±232.1 (160–1195)	57-54-14	152	71	37±17.5 (6–94)	5±6.1 (0–27)
**Restricted set of reserves**								
Plants	97	78±74.9 (20–348)	392±126.4 (150–650)	27–64-5	1577	664	204±102.7 (38–476)	32±32.7 (0–165)
Butterflies	47	120±97.0 (25–350)	381±143.6 (160–650)	20–25-2	136	56	42±21.8 (9–94)	7±7.6 (0–94)

1) Numbers of sites belonging to thermophyticum (T), mesophyticum (M) and oreophyticum (O) regions, defined by combining topography and climate.

#### Butterflies in reserves

Surveys, restricted to 125 National reserves (Fig. 1), were carried out in 2004–2006 [30]. Targeting butterflies, reserves protecting homogeneous woodlands expectably poor in butterflies were underrepresented at the expense of grasslands. Each reserve was assigned to a lepidopterist, who visited it five times between May and September, always under suitable weather, each time checking all biotopes present and following an approximately identical path, but paying particular attention to seasonally changing locations of such butterfly resources as nectar [31]. Visit durations scaled with reserve area (<25 ha: 1 h, <50 ha: 1.5 h, <100 ha: 3 h, <200∶4 h, above 200 ha: 5 h).

#### Plants in military areas

The 42 medium-sized MTAs surveyed constitute all such sites that were historically used by armoured army units, and until now were not completely build-up, afforested, or turned to arable land. They are distributed evenly across the country (Fig. 1) and in terms of area and altitude, they are more homogeneous than the reserves surveyed for plants and butterflies (Table 1). Species lists were compiled during intensive surveys by two botanists in summer 2008, visit durations again scaled with area (<25 ha: 3h, <50 ha: 4 h, <100 ha: 8 h, <200 ha: 10 h, <200 ha: 12 h) and covered all habitat types present in each site.

#### Butterflies in military areas

Following the identical procedure as for butterflies in reserves, 41 sites were inventoried in 2008.

### Variables and Analyses

*Plant* (species) *richness*, *Endangered plants*, *Butterfly* (species) *richness*, and *Endangered butterflies* were response variables, whereas site *STATUS* – Reserve *vs.* MTA – was the main factor of interest.

To compare the species richness patterns, we employed a regression approach aiming on statistically controlling confounding effects of unequal sample sizes and the sites’ geographic position, area and climate on the response variables. For each site, we considered the following readily available characteristics, likely influencing local species richness: Area; Latitude (Lat); Longitude (Long) and Altitude (Alt), all standing for geography patterns affecting species’ distributions; Altitude range (ARang), a proxy of topographic and mesoclimatic heterogeneity; and Phytogeography region (Veg), based on the division of the Czech territory into thermophyticum, mesophyticum and oreophyticum, according to a combination of topography and climate. We coded Veg as a 3-levelled ranked variable, expecting the highest species richness in the warmest thermophyticum [29].

Using the generalised linear modelling in R [32] and assessing the models following the information theory approach (*AIC* values), we first ran single-predictors’ tests with *STATUS* against the four response variables. We then computed, separately for each response, single-terms’ regressions with all possible covariables, including their second-degree polynomials, to check the directions and strengths of the responses. Next, we constructed fully saturated models containing all potential covariables (in the forms suggested by the single-term tests) and their interactions (up to 2nd-order). We simplified these models using the R backward-elimination procedure, until we obtained minimal adequate models (*MAMs*) containing only predictors that improved the respective models fits without introducing unnecessary complexity. *MAMs* thus represented the best explanations of the response variable distribution using the covariables considered, and hence a maximum possible statistical control for the covariables’ nuisance effects. Finally, we added the categorical predictor *STATUS* onto the *MAMs*, and compared these *MAM-STATUS* models with *MAM* models. If *MAM-STATUS* and *MAM* differed by ΔAIC ≤2.0, we considered it as improving the fit. These comparisons assessed the effect of reserve *vs*. military area on response variable after considering the effects of all covariables.

*Plant richness*, *Endangered plants* and *Butterfly richness* data were analysed with the Gaussian distribution of errors, following logarithmic (*Plant richness*, *Endangered plants*) and square-root (*Butterfly richness*) transformations. *Endangered butterflies* data were modelled with the Poisson error distribution.

We subsequently repetated the entire regression procedure for a subsample of reserves with areas (20–355 ha) and altitudes (150–650 m) matching those of the MTAs. This provided a more direct comparison between reserves and MTAs that were comparable natural conditions. Total of 97 reserves fulfilled the selection criteria for plants, 47 reserves fulfilled them for butterflies (Table 1).

To compare the reserves and MTAs with respect to species composition, we used Canonical correspondence analysis (CCA), a unimodal ordination method that relates the species composition of samples to the samples’ environmental characteristics. We carried out the analyses in CANOCO for windows [33], using the Monte Carlo tests (999 permutations under reduced model) to asses the significance of ordination results. Individual localities were samples in these analyses, species records formed the species data matrix. As in the regressions, we first related the species composition to *STATUS* only. Second, to account for variation in environmental conditions, we built covariate models based on forward selection of site characteristics, their polynomial terms and second-degree interactions. Finally, again following the procedures used for the univariate regressions, we tested for the partial *STATUS* significance after including the forward-selected covariate terms onto the models, thus asking if *STATUS* explained some additional variation in species composition of the samples.

To interpret the ordination results, we used a simple metrics, the relative representation of endangered species among the species most tightly associated with extreme of the canonical axis, i.e. with reserves *vs*. MTAs, in the CCA models containing *STATUS* and covariates. For plants, we considered 100 species at each side of the gradient, i.e., 100 species most tightly associated with reserves and 100 species most tightly associated with MTAs; these 200 species corresponded to 10 percent of plant species analysed (cf. Table 1). For butterflies, with considerably fewer species in total, we considered the upper and lower quartiles along the canonical axis (or 50% of all species analysed), amounting 38 species at each side.

## Results

The reserves contained 1941 plants species (884 endangered), and 152 butterfly species (71 endangered). The MTAs contained 873 plant species (160 endangered), and 118 butterfly species (42 endangered) (Table 1). The reserves thus harboured a majority of the Czech Republic flora and butterfly fauna, and the MTAs harboured a tenth of red-listed vascular plants species and a half of red-listed butterflies.

Not controlled for covariables, MTAs contained higher *Plant richness* than reserves (Mann-Whitney U-test: *z* = −2.71, *P*<0.01), hosted fewer *Endangered plants* (*z* = 3.00, *P*<0.01), higher *Butterfly richness* (*z* = −4.84, *P*<0.0001), and did not differ in numbers of *Endangered butterflies* (*z* = −0.29, *P* = 0.79) (Table 1).

In the single-term regressions (Table 2), most of the potential covariables influenced the responses. *Plant richness*, *Endangered plants* and *Endangered butterflies* responded to the predictors more tightly, in terms of the number of predictors reducing the original data deviation, than *Butterfly richness*. None of the two butterfly responses were affected by site Area, and *Butterfly richness*, contrary to *Endangered butterflies*, did not respond to Latitude, Longitude or Altitude range. For all four responses, polynomial models frequently achieved better fits than linear models.

**Table 2:** Results of single terms regressions showing the relationships of response variables to predictors, subsequently used as covariates in the minimum adequate models (*MAM*) comparing Czech Republic reserves and military training areas.

	Plant richness			Endangered plants			Butterfly richness			Endangered butterflies		
	Deviance	AIC	b	Deviance	AIC	b	Deviance	AIC	b	Deviance	AIC	b
**All reserves analysed**												
Null	27.2	107.3		94.0	534.6		306.8	577.0		895.7	1364	
Area	26.1	95.1	↑^ L^	88.4	515.2	↑	306.2	578.7	–	895.6	1366	–
Lat	27.2	108.9	–	92.9	534.3	–^P^	300.9	577.8	–^ P^	814.1	1287	↓↑
Long	26.2	98.1	↑↓	93.9	536.1	–	303.3	577.1	–	860.5	1333	↑↓
Alt	24.7	78.7	↓↑	90.6	525.8	↓↑	273.2	561.8	↓↓	833.7	1307	↓↓
ARang	26.6	101.4	↑	88.3	514.8	↑	305.2	578.2	–	783.6	1254	↑
Veg	25.3	84.2	↑	91.3	526.4	↑	279.2	563.4	↑	863.5	1334	↑
**Restricted set of reserves analysed**												
Null	1376.2	721.3		31.9	194.5		162.8	307.9		508.5	775.4	
Area	1367.7	722.4	–^ L^	31.9	196.3	–^L^	162.4	309.5	–^L^	505.4	774.3	↑
Lat	1368.1	722.4	–	31.8	195.9	–	156.9	308.6	–^ P^	429.9	700.8	↓↑
Long	1345.1	722.1	–	31.8	195.8	–	162.8	309.9	–	471.3	740.2	↑
Alt	1320.5	719.5	↓↓	31.5	194.3	↓	143.5	300.8	↑↓	458.9	729.8	↓↓
ARang	1368.5	722.5	–	31.4	194.0	↑	150.6	303.0	↑	421.7	690.6	↑
Veg	1353.9	721.0	↑	30.7	190.9	↑	159.6	308.1	–	467.4	736.4	↑

*AIC*-Akaike information criterion; *b* – the darts indicate directions of the relationships, ↑standing for increasing, ↓ for decreasing, ↑↓ for polynomial trends with darts indicating the fitted functions directions. Upper-case ^P^ indicates a situation when polynomial performed better than linear trend, but still did not improve the model. Similarly, Upper-case ^L^ indicates a situation when log10-transformed predictor performed better than untransformed predictor, but still did not improve the model.

The predictors were: Area, Latitude (Lat), Longitude (Long), Altitude (Alt), Altitude range (ARange) and Phytogeography region (Veg; a ranked variable, from coldest to warmest region of the country).

**Figure 2:**
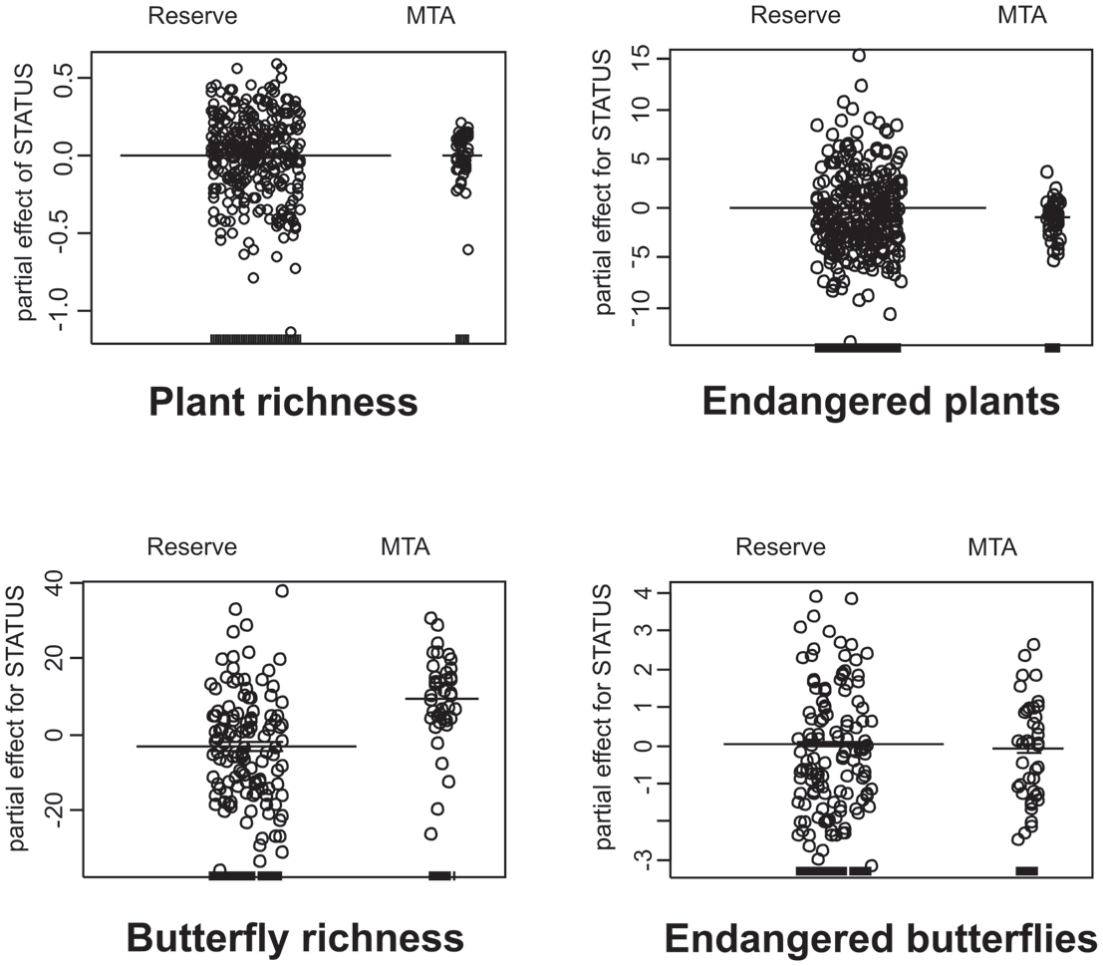
Effects of site status on species richness, and numbers of red-listed species, of vascular plants and butterflies, in Czech Republic reserves and military training areas: partial effects of site status from models containing all other significant covariables.

The variation explained by the minimum adequate models (*MAM*s: Table 3) ranged between 15.1% (*Endangered plants*) and 41.6% (*Endangered butterflies*). Adding the reserve *versus* MTAs predictors (*MAM-STATUS* models: Table 3 and Fig. 2) did not improve the fit for *Plant richness*, implying no difference between reserves and MTAs. More *Endangered plants* occurred in reserves, higher *Butterfly richness* existed in MTAs. Finally, MTAs and reserves hosted equal numbers of *Endangered butterflies*.

**Table 3:** Summary of GLM regressions used to compare species richness, and the numbers of endangered species, in the Czech Republic reserves and military training areas (MTAs).

Model	Model terms1)	*d.f.*	*Residual deviance*	*Fitted* *deviance*	*AIC*
**All reserves analysed**					
***Plant richness***					
*Null*		343	27.2		107.3
*STATUS*	**Reserves<MTAs**	1, 342	26.8	0.014	104.2
*MAM*	+L(Area) ±Alt +Veg -ARang+(L(Area)*ARange)	6, 337	21.8	0.198	43.9
*MAM-STATUS*	**Reserves ≈ MTAs**	7, 336	21.8	0.198	45.9
***Endangered plants***					
*Null*		343	94.0		534.6
*STATUS*	**Reserves>MTAs**	1, 342	93.0	0.012	532.6
*MAM*	+Area +Lat +Long +(Lat*Long) +ARang +Veg	6, 337	79.9	0.151	490.2
*MAM-STATUS*	**Reserves>MTAs**	7, 336	77.6	0.175	482.4
***Butterfly richness***					
*Null*		165	306.8		577.0
*STATUS*	**Reserves<MTAs**	1, 164	276.0	0.100	561.5
*MAM*	+Long ±Alt +ARang +Veg	5, 160	235.0	0.234	542.8
*MAM-STATUS*	**Reserves<MTAs**	6, 159	210.5	0.314	526.5
***Endangered butterflies***					
*Null*		165	895.7		1364.1
*STATUS*	**Reserves>MTAs**	1, 164	887.8	0.009	1359.0
*MAM*	+Lat ±Alt ±Long +ARang +Lat*Long +Veg	8, 157	522.7	0.416	1007.3
*MAM-STATUS*	**Reserves ≈ MTAs**	9, 156	530.0	0.408	1013.1
**Restricted set of reserves**					
***Plant richness***					
*Null*		139	1376.2		721.3
*STATUS*	**Reserves ≈ MTAs**	138	1374.9	0.001	723.1
*MAM*	±Alt	2, 137	1320.5	0.040	719.5
*MAM-STATUS*	**Reserves ≈ MTAs**	3, 136	1316.7	0.043	721.1
***Endangered plants***					
*Null*		139	31.9		194.5
*STATUS*	**Reserves>MTAs**	1, 138	28.6	0.106	180.8
*MAM*	+ARang +Veg	2, 137	30.1	0.059	189.9
*MAM-STATUS*	**Reserves>MTAs**	3, 136	25.9	0.190	170.9
***Butterfly richness***					
*Null*		87	162.8		307.9
*STATUS*	**Reserves<MTAs**	1, 86	152.4	0.064	304.0
*MAM*	±Alt +ARang	3, 84	138.8	0.147	299.8
*MAM-STATUS*	**Reserves<MTAs**	4, 83	129.0	0.208	295.5
***Endangered butterflies***					
*Null*		87	508.5		775.4
*STATUS*	**Reserves>MTAs**	1, 86	474.6	0.067	743.5
*MAM*	**±**Lat +Long **±**Alt +Veg +(**±**Lat*Long)	8, 79	233.8	0.540	516.7
*MAM-STATUS*	**Reserves>MTAs**	9, 77	220.7	0.566	505.6

1) Terms of the models, see Material and methods for abbreviations. ±sign stands for second-order polynomial.

*STATUS* models refer to effects of reserve vs. MTA without control for covariables, *MAM* models include a combination of all covariables and interactions whose effect differed from zero and from one another, whereas *MAM-STATUS* models asses the effect of reserve *vs*. MTA on residuals from *MAM* models.

In the single-term regressions of the subsample of reserves comparable in size and altitude to the MTAs, notably fewer predictors fitted the data for *Plant richness* and *Endangered plants* than for *Butterfly richness* and *Endangered butterflies* (Table 2). Multiple regressions (Table 3) showed that *Plant richness* was identical in reserves and MTAs, even according to *MAM-STATUS* model. *Endangered plants* were more numerous in reserves, again also in *MAM-STATUS* model. The opposite applied for *Butterfly richness*, which was higher in MTAs. The pattern differed from the regressions considering all reserves for the case of *Endangered butterflies*, which were now more numerous in reserves.

CCA ordinations pointed to highly significant differences in species composition of both plant and butterfly assemblages attributable to *STATUS*, even after controlling for covariate effects (Table 4). The proportion of variation in species data attributable to *STATUS* was coniderably higher for plants than for butterflies (see the Axis 1 eigenvalues in Table 4), but did not drop too markedly after inclusion of covariates, indicating that some species tended to occur in reserves, and some in MTAs, independently on geographic position and topography of the sites.

**Table 4:** Results of Canonical correspondence analyses comparing the plant and butterfly species coposition recorded in nature reserves and military training areas of the Czech Republic.

	Axis 2	Axis 2	Axis 3	Axis 4	Summed eigenvalues	Axis 1: *F, P*	All axes: *F, P*
**Plant species composition**							
*∼STATUS*	0.140	0.405	0.257	0.247	8.723	5.586***	
*∼COVARIATES*	0.132	0.102	0.056	0.050	8.723	5.100***	2.089***
*∼STATUS|COVARIATES*	0.123	0.333	0.236	0.216	8.162	5.057***	
**Butterfly species composition**							
*∼STATUS*	0.037	0.228	0.174	0.137	2.617	2.376***	
*∼COVARIATES*	0.170	0.079	0.076	0.053	2.617	10.707***	3.508***
*∼STATUS|COVARIATES*	0.033	0.116	0.111	0.079	2.093	2.473***	

Selected plant *COVARIATES*: ∼log(Area) +Long^2^+Lat^2^+Alt^2^+Arange +Veg +Long*Lat +log(Area)*ARange.

Selected butterfly *COVARIATES*: ∼Area +Lat^2^+Long^2^+Al^2^+Lat*Long +ARange +Veg +Area*ARang.

*STATUS* models are directly comparing the two land use caterogires, *COVARIATES* models were constructed by a forward selection of site characteristics potentially influencing the species composition, whereas *STATUS|COVARIATES* models are testing the marginal influence of *STATUS*, after fitting the *COVARIATES* terms into the models. *F* and *P* values refer to the Monte Carlo permutation tests.

Potential covariates were: site Area, latitude (Lat), longitude (Long), Altitude (Alt), Altitude range (ARange) and Phytogeography region (Veg; a ranked variable, from coldest to warmest).

Among the 100 plants most markedly associated with reserves (Table S2), there were 76 endangered species, whereas among the 100 plants most markedly associated with MTAs, there were 27 endangered species. This disproportion corroborated the above observation of higher representation of *Endangered plants* in reserves, but in the same time revealed that some endangered plants were relatively overrepresented in MTAs. A cursory examination of the oridination results suggested that reserves hosted predominately species associated with rare habitats such as high mountains (e.g., *Poa riphaea*, *Tophieldia calyculata*), wetlands (e.g., *Pinguicula bohemica*, *Euphorbia palustris*) or warm grasslands (e.g., *Amygdalus nana*, *Rosa micrantha*), whereas the endangered plants associated with MTAs were species of disturbed grounds (e.g., *Corynephorus canescens*, *Equisetum hyemale*, *Dorycnium herbaceum*) or woodland edges (e.g., *Dispacus lacinatus*, *Lathyrus hirsutus*). Notably, these species were accompanied by numerous invasive alliens (*Acer negundo*, *Rudbeckia hirta*, *Solidago gigantea*).

Regarding butterflies, the group of 38 species associated with reserves contained 33 endangered species, whereas the equally large group associated with MTAs contained 13 red-listed species (Table S2). As in the case of plants, the endangered butterflies associated with reserves were often species with narrow biotope requirements and correspondingly narrow distribution ranges in the country (e.g., peat bog species *Vacciniina optilete*, *Coenonympha tullia*, or southern limits species *Neptis rivularis*, *Brenthis hecate*). The group associated with MTAs was rather heterogeneous, encompassing species of open woodlands (e.g., *Hipparchia fagi*), abandoned grasslands and scrub (e.g., *Zygaena brizae*, *Arethusana arethusa*, *Minois dryas*) and species associated with baren ground (e.g., *Polyommatus bellargus*, *Polymmatus dorylas*).

## Discussion

While conservation community increasingly accepts the biodiversity value of large and actively used military training ranges [13], [25], [34], and armies of developed countries increasingly participate in conservation efforts [11], [12], until recently there was no systematic interest in numerous small MTAs, which are being abandoned in recent decades. Our sample of 43/41 Czech Republic MTAs harboured 160 nationally threatened plants and 42 nationally threatened butterflies. It matched a representative sample of the country reserves in *Plant richness* and exceeded it in *Butterfly richness*. On the other hand, the MTAs hosted fewer *Endangered plants* than did nature reserves, while an ambiguous pattern applied for *Endangered butterflies*. Different sets of species inclined towards reserves than towards MTAs, which implies that the reserves and the sites once used by military harbour somehow different segments of the nation’s biodiversity.

Plant surveys in reserves were in fact more comprehensive (carried out by local botanists, based on multiple visits) than the plant surveys in MTAs. This should have favoured the reserves over MTAs, but despite this, *Plant richness* in MTAs and reserves did not differ. Some bias towards reserves also affected the butterfly data. The reserves butterfly survey targeted the most valued reserves in the country, deliberately excluding woodlands presumably poor in butterflies [30]. Still, *Butterfly richness* in MTAs exceeded that in the reserves. Finally, although the numbers of reserves surveyed differed between plants and butterflies, both samples of sites were distributed evenly across the country, covering similar ranges of area and altitude.

The high representation of *Endangered plants* in reserves is easily explained, given the history of the Czech Republic reserve network. Due to a traditional pivotal position of botanists in national conservation, a majority of reserves was established with plant conservation in mind [29] so that a high representation of endangered plants constitutes a defining feature of many reserves. In addition, Central European conservation was much influenced by the Zürich-Montpellier phytosociology [35], emphasising the conservation of representative plant communities. Boundaries of many reserves, especially the small ones, then often copy the boundaries of the targeted plant communities, possibly again increasing the relative representation of threatened plants on the expense of non-threatened ones [36]. The richest rare plants localities already enjoyed legal protection in the pre-WWII period [37] and were unlikely appropriated by the military, whereas MTAs were typically carved out from more ordinary rural landscapes. The ordination analysis also corroborated that plants most prominently inclining towards reserves were rarities of such nationally rare habitats as alpine grasslands, where early protection status excluded a military use.

The patterns found for *Endangered butterflies* differed between the analysis considering all 125 reserves surveyed and the analysis restricted to the 47 reserves of areas and altitudes comparable with the MTAs. As in the case of *Endangered plants*, this restricted analysis suggested that reserves, rather than MTAs, tended to host endangered buterflies. Again, a typical reserve protects a rare or declining habitat and the butterflies associated with the reserves were specialists of such habitats as oligotrophic wetlads, eutrophic wetlands, or warm grasslands. Still, some rare habitats are notoriously poor with butterflies [38] but contain multiple red-listed plants [39]. The Czech Republic examples include salt marshes and sand dunes, preserved only in a handful of tiny reserves, or subapline grasslands, protected in a few, but usually large reserves. Removing the smallest plus the largest reserves from the analyses affected such butterfly-poor habitats dispropotionatelly, causing the disparity between *Endangered butterflies* and *Endangered plants* results.

The higher *Butterfly richness* in MTAs clearly demonstrated the value of such sites for conserving biodiversity, while supporting our initial conjecture regarding the value of distrubances, and ensuing habitat mosaics, for high species richness. A great number of European butterflies, as well as insects from other groups, thrive in heterogeneous habitat mosaics, requiring diverse resource located in close proximity [22], and heterogeneity promotes population stability [40]. Some species may be even suppressed from some reserves by too meticulous management approches, which strive to maintain representative examples of specific plant communities on the expense of edges, transient zones and successional stages. Techniques such as mowing and grazing, while necessary for blocking succession, may, if applied insensitively, decrease the local insect richness via direct mortality [41], [42] or periodic resource depletions [43], [44], [45].

The role of heterogeneous disturbance-succession dynamics for MTAs butterflies was futher corroborated by ordination analysis. Although more endangered butterflies inclined towards reserves, some inclined towards MTAs, and these were either species requiring disturbed ground, or species thriving in abandoned grassland and scrub. Sizeable patches of disturbed grounds still exist in majority of the MTAs surveyed as a legacy of past military activities, currently maintained by (semi-illegal) motocycling and off-road driving. They are rateher rare, however, in reserves, because managing agencies often hesitate with applying more drastic management approaches. Grasslands, on the other hand, are often too meticulously managed in reserves, in contrast to MTAs.

Similar arguments as for MTAs *Butterfly richness* likely apply for MTAs *Plant richness*, and the potential role of military-affected lands for plant conservation in general. MTAs were favoured, as in the case of butterflies, by plants growing at disturbed grounds, and by plants of woodland edges and open woodlands, i.e., succesionally transient habitats. This picture is somehow blurred, however, by many invasive alliens strongly inclining towards MTAs. Many alliens in Central European flora establish readily at disturbed grounds [46], where they may compete with some distrurbance-dependent rare species. This further highlights, however, the value of the disturbance-succession mosaics characteristic for past military use, because small-scaled patchiness arguably promotes the coexistence of multiple plants, including poor competitors, in close proximities [25], [47].

The main message from this study is that not only large army training ranges covering hundreds of square kilometres [11], but also relatively small MTAs covering a few dozens of hectares, harbour species numbers matching, or surpassing, the purportedly richest biodiversity localities protected in nature reserves. The high species richness is attributable to heterogeneous distrubance-succession mosaics, created and maintained by military activities. Regarding endangered species, those characteristic for MTAs are either species of barren surfaces [13], [24], [47], directly depending on mechanical disturbances, or species of neglected grasslands, edges and transition zones, benefiting from the diverse successional conditions [16]. Both diverse disturbance events and subsequent heterogenous conditions were common in pre-intensification cultural landscapes, from which most the MTAs were carved more than half century ago, but they are underrepresented both in the established reserves and in modern cultural landscapes [34]. As a result, both reserves managed for preselected plant communities [36], and increasingly uniformised agriculture and forestry [44] fail to sustain many species still thriving in MTAs. A secondary message is that management of many reserves may need a reconsideration towards maintaining a greater diversity of successional stages, if the full biotic potential of already protected sites is to be utilised [48].

Small to medium-sized military areas, many of them recently abandoned by the armies, represent a priceless biodiversity conservation opportunity. Cessation of military use is threatening these sites either by development, or, alternatively, by successional homogenisation of the currently diverse habitat mosaics. Across Europe, and probably elsewhere, the conservation value of many such sites may soon be lost, if they are not exempt from building development, and if appropriate disturbance regimes are not provided. Pragmatic options to replace the armies include various sporting activities (four-wheel driving, horse-riding etc.), or reestablishment of large herbivores, both having the potential to maintain biotic richness of the sites while supplying other public goods.

## Supporting Information

Table S1 List of reserves and MTAs surveyed for plants and butterflies, including numbers of species recorded and basic site characteristics. Sheet 1: Plant sites, Sheet 2: Butterfly sites.
(XLS)

Table S2 Plant and butterfly species most tightly associated with the Czech Republic nature reserves *versus* military training areas, according to the canonical correspondence analysis controlled for covariables characterising individual sites. Endarngered species are listed in ***bold letters***. Sheet 1: Plant sites. Sheet 2: Butterfly sites.
(XLS)
